# Career Future Time Perspectives, Social Media Engagement, and the School-to-Work Transition in Emerging Adulthood

**DOI:** 10.3390/bs16040506

**Published:** 2026-03-28

**Authors:** Katrin Kreutz

**Affiliations:** Department of Education, Friedrich-Alexander-University Erlangen-Nuremberg, 90478 Nuremberg, Germany; katrin.kreutz@fau.de

**Keywords:** emerging adulthood, social media, problematic social media use, transition orientation, moratorium orientation, future time perspectives

## Abstract

This study investigates the relationship between general and problematic social media use, and young adults’ future time perspectives during their school-to-work-transition. It also explores how parents perceive the influence of their children’s media use on career perspectives. Utilizing longitudinal data from a quantitative study, 443 parent–youth dyads at t1 and 355 at t2 were surveyed on their practices concerning daily social media use, problematic social media engagement, transition and moratorium orientations, and parental assessments. Open-ended responses from parents indicated that the majority perceived either a positive effect or no influence of media use on career opportunities, while a smaller proportion reported negative impacts. Adolescents whose parents expressed positive views demonstrated significantly stronger transition orientations. Cross-sectional analyses demonstrated that problematic social media use was negatively associated with transition orientation and positively related to moratorium orientation. General usage time, however, showed no meaningful associations. Longitudinal regression analyses indicated that neither general nor problematic social media use predicted subsequent levels of transition or moratorium orientation after controlling for baseline orientations, pointing to substantial stability in these dispositions. The findings suggest that problematic social media engagement coincides with less future-oriented mindsets, while future orientations remain stable over time.

## 1. Introduction

Emerging adulthood represents a critical developmental period in which, among other important tasks, individuals navigate the transition from educational organizations to employment. This process is profoundly shaped by temporal orientations toward the future (Arnett, 2007). Future time perspective, understood as individuals’ perceptions, motivations, and expectations regarding their remaining time, has been identified as a key determinant of decision-making, goal-setting, and long-term developmental outcomes such as career success and well-being (Cate & John, 2007; Zimbardo & Boyd, 1999). As a multifaceted human trait, the concept of future time perspectives facilitates adaptation across life domains, especially during life transitions, such as the time of emerging adulthood (Poletti et al., 2023). Steinberg et al. (2009) demonstrated that, for young adults, orientation assumes greater significance for the future from the age of 16 onwards. In their systematic review with a meta-analysis, Kooij et al. (2018) found that the concept of future time perspective has, on the one hand, been largely under-researched to date and, on the other hand, is defined very differently depending on the methodology used, and that it has various connections with personality and psychological characteristics. In previous literature, it has been demonstrated that individuals who possess a more expansive future time perspective are characterized by the presence of clearly defined goals, the enhancement of their planning skills, and an augmented capacity to effectively cope with future challenges (Basilici et al., 2025). Furthermore, extant research has demonstrated a correlation between unemployment and adverse future time perspectives (Parola et al., 2023). Nonetheless, the future time perspective of young adults is not a standalone phenomenon depending on individual factors (Mello & Swanson, 2007; Mello & Worrell, 2015); rather, it is contingent on social factors. For example, socio-economic status, parental home and parenting all exert a decisive influence on the shaping of this perception (Calmbach & Schleer, 2020; Parola et al., 2023; Seginer, 2009; Timar-Anton et al., 2023).

As indicated by extant literature on the subject, career planning is one of the five domains identified in future time perspectives (Koposko & Hershey, 2016). This is an integral component of the emerging adulthood phase and the transition from school to the working world. In the contemporary discourse on emerging adulthood, a notable emphasis has been placed on the notion of a moratorium. This moratorium orientation—mostly in delineation to transition orientation—is characterized by a protracted or deferred decision-making phase regarding one’s own career development trajectory (Arnett, 2007; Reinders, 2005). Consequently, decisions regarding further education, university studies, or the commencement of a career are deferred, resulting in an absence of orientation towards future career aspirations.

Contemporary social developments further underscore the tendency among young individuals to invest a greater amount of time in exploring potential career pathways (Arnett, 2007; Reinders, 2005). This has the effect of prolonging the period of moratorium, as conceptualized by Erikson (Erikson, 1968; Valkenburg & Piotrowski, 2017). Increasingly, this transition is embedded within a deep mediatized society (Hepp, 2019) in which media plays a central role in shaping both opportunities and challenges for young adults’ career trajectories. In particular, social media has been identified as a significant influence in this regard, given the extensive range of career paths and job realities that young people encounter through their interactions on such platforms (Jahncke et al., 2020; Uhls et al., 2017). The role of social media in this context is not considered to be the primary influencing factor on future time perspectives; rather, it is viewed as a component of a multifaceted construct encompassing individual, social, and media factors that can influence the transition of young people from school to the working world (Uhls et al., 2017). While general social media use can provide informational, networking, and identity-related resources, problematic use refers to maladaptive patterns characterized by excessive time investment, loss of control, preoccupation, and the persistence of use despite negative consequences for daily functioning (Andreassen, 2015; Shannon et al., 2022). The repercussions of such a loss of control can also manifest in the neglect of school and work responsibilities which may be associated with difficulties in managing school and work responsibilities and may therefore relate to challenges during the transition into working life. In recent years, problematic social media use has been conceptualized in a manner analogous to problematic gaming which has already been included in the International Classification of Diseases (ICD-11 classification) of the World Health Organization (WHO) (World Health Organization, 2018). However, additional research on problematic social media has gradually gained importance, often focusing on the target group of adolescents and young adults (Cheng et al., 2021). This is attributable to the fact that young people are particularly affected by problematic social media use because of developmental factors (Montag et al., 2024). Estimates of prevalence rates for German adolescents between the age of 14 and 17 years in 2024 suggest that approximately 5.4% are affected by problematic social media use (Wiedemann et al., 2025).

Drawing upon these developments, the present article focuses on the connection between (problematic) social media use and future time perspectives in relation to young adults’ transition from school to working life. The analysis incorporates parents’ assessments of the influence of digital media on their children’s career opportunities.

## 2. Conceptual Framework

**Figure 1:**
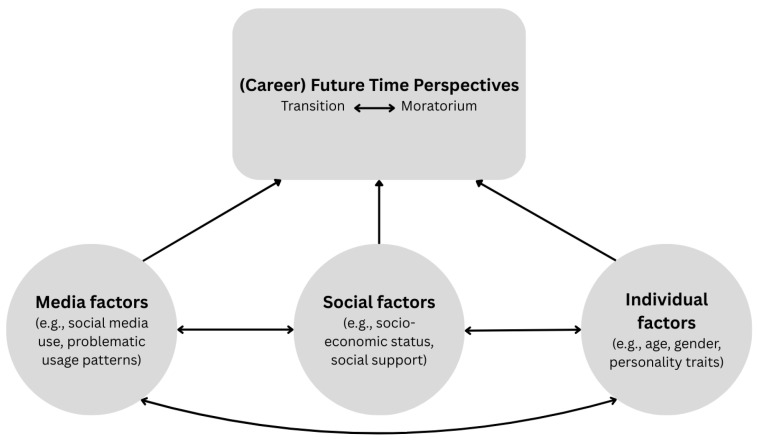
Conceptual framework linking future time perspective to individual, social and media influencing factors.

In order to investigate the relationship between future time perspective and social media use, the present study builds on existing research by adopting an integrative conceptual framework that considers social media use in the context of broader developmental processes shaping the transition from school to work in emerging adulthood. In light of the paucity of extant models that adopt a more expansive perspective on the notion of future time perspective and the interplay between diverse influencing factors (Fang & Saks, 2022; Kooij et al., 2018), a dedicated overview has been devised for the purpose of elucidating these concepts. Figure 1 illustrates schematically that (career-related) future time perspectives are an individual varying construct influenced by various factors. The model emphasizes individual and social factors, which have previously been identified as important predictors on future time perspectives, as well as the influence of the media, such as the use of social media, in the context of a deep mediatized society. The framework is grounded in a life-span developmental perspective that emphasizes the role of enduring, future-oriented dispositions. The framework delineates the interconnection of individual, social, and media factors that influence future time perspectives, thereby demonstrating their mutual influence. This is in contrast to conceptualizing digital media use as a primary causal driver of transition outcomes. The notion of future time perspective occupies a pivotal position within this theoretical framework. The orientation towards transition and moratorium is regarded as an integral component of the future time perspective in the context of career development and seen as a domain-specific manifestation of career-related future time perspective.

Within the school-to-work transition, future time perspective is reflected in more domain-specific orientations. The concept of orientation towards transition captures a future-oriented and goal-directed mindset, characterized by planning, commitment, and confidence in achieving occupational goals. Conversely, the concept of orientation towards moratorium reflects a present-focused orientation, marked by postponement of binding decisions and openness towards alternative life options (Reinders, 2005). Moratorium is understood as a historically established feature of identity development that predates contemporary digital media environments (Erikson, 1968). Consequently, both orientations are conceptualized as enduring developmental dispositions rather than as outcomes primarily produced by social media use. Social media use is therefore positioned in this framework as one influencing factor among others within a deep mediatized society (Hepp, 2019), rather than as a primary causal driver of transition-related orientations. A crucial distinction is made between general social media use, operationalized as usage time, and problematic social media use, which refers to maladaptive engagement patterns characterized by loss of control, excessive involvement, and negative consequences for everyday functioning (Andreassen, 2015; Cheng et al., 2021).

When considered as a whole, this theoretical framework positions the use of social media as a component of a multifaceted developmental process, in which enduring future-oriented dispositions, contextual digital practices, and social evaluations collectively influence the school-to-work transition in emerging adulthood.

## 3. State of Research

As derived from the conceptual framework, future time perspectives and the transition from school to working life are influenced by a multitude of individual, social, societal, and media factors (e.g., Böhle et al., 2025; Kooij et al., 2018; Parola et al., 2023). This paper and the following overview on the state of research explicitly focusses on the connection to media use, social media use as well as problematic usage patterns.

While general use has been shown to provide informational, social, and identity-related resources that are relevant to career development (Uhls et al., 2017), problematic social media use is assumed to be more closely associated with difficulties in self-regulation and a stronger focus on immediate gratification (San Martín Iñiguez et al., 2024). This distinction is crucial, as maladaptive engagement patterns have repeatedly been linked to adverse psychosocial outcomes, including increased distractibility, impaired self-regulation, and reduced goal-directed behavior, which may undermine young adults’ capacity to plan and pursue long-term goals.

Problematic engagement with social media has repeatedly been associated with adverse psychosocial outcomes, including increased distractibility, impaired self-regulation, and reduced goal-directed behavior. These outcomes may, in turn, undermine young adults’ capacity to plan and pursue long-term goals. As Kaya et al. (2025) demonstrated in their explorative study, problematic social media use has been associated with difficulties in decision-making via ruminative identity exploration. A growing body of research has examined the relationship between digital media use and psychosocial development more broadly, demonstrating both risks and resources associated with online engagement (for example, see the review by Senekal et al. (2023)). However, despite the expansion of the relevant literature, empirical research explicitly linking social media use to the transition from education to employment remains scarce. The extant literature indicates that the function of social media in educational and transitional contexts is multifaceted rather than unidirectional. Dennen et al. (2024) highlighted that social media can facilitate access to information, peer support, and networking opportunities during the transition from high school to university. However, they also identified risks related to well-being, distraction, and social isolation. Zhang et al. (2024) found a more career-oriented social media use as an increasing factor for career anxiety in turn promoting stronger career exploration for Chinese university students. In addition, early empirical evidence indicates that problematic digital behaviors are related to temporal orientations. Research by Przepiorka and Blachnio (2016) was among the first to identify a negative correlation between a future time perspective and problematic internet or Facebook use, suggesting that individuals with a less future-oriented outlook may be more vulnerable to maladaptive digital behaviors. In relation to extreme media usage, there have been some findings showing a negative influence on the school performance of adolescents (Díaz-López et al., 2021; OECD, 2016, 2017; Wallner-Paschon et al., 2018), which in return could lead to problems in the school-to-work-transition. Moqbel and Kock (2018) examined in their study that an addiction to social networking sites has negative consequences for personal and work environments, especially due to the issue of task distraction.

In view of the dearth of studies with a specific focus on social media, insights are frequently drawn from related research on problematic gaming, internet use, or smartphone addiction. These phenomena are often conceptualized as being interconnected. Within this broader field, several studies have indicated a protective role of a positive future time perspective. As posited by Dou et al. (2022), an expansive future orientation was found to be negatively associated with excessive gaming. In contrast, Díaz-Aguado et al. (2018) discovered that maladaptive future time perspectives were linked to problematic gaming among Spanish adolescents. The evidence of scarce longitudinal studies provides further support for this association: As demonstrated by Shan et al. (2023), a positive future time perspective has been shown to function as a negative predictor of gaming disorder over time. Conversely, research conducted with Chinese adolescents suggests that future expectations may act as a protective factor against internet addiction (Du & Lyu, 2021). However, findings are not entirely consistent across different forms of problematic media usage patterns. For instance, Li et al. (2024) reported no significant association between future time focus and smartphone addiction, underscoring the importance of differentiating between types of digital engagement and outcomes.

A synthesis of extant literature suggests a relationship between future-oriented dispositions and problematic forms of digital media use; however, the evidence remains fragmented. It should also be considered that problematic social media use and moratorium-oriented mindsets may not necessarily be linked through a unidirectional causal pathway. Both constructs may reflect underlying self-regulatory or developmental processes that simultaneously influence digital engagement patterns and future-oriented dispositions. From this perspective, problematic use may function less as a primary cause and more as an indicator of broader regulatory or motivational dynamics relevant to the school-to-work transition. The findings emphasize the necessity for a diversified perspective that transcends simplistic assumptions concerning media effects, instead focusing on the quality and regulation of individual digital or social media engagement. A paucity of longitudinal studies has been observed, particularly in the integration of future time perspectives with domain-specific orientations relevant to the school-to-work transition, such as transition and moratorium orientations. Extant empirical findings suggest that problematic social media use is associated with a stronger moratorium orientation and a weaker transition orientation. General usage time is expected to play a subordinate role. These relationships will be analyzed as temporal predictions reflecting concurrent associations between digital engagement patterns and future-oriented dispositions. Furthermore, in accordance with preceding research on future time perspective, transition- and moratorium-oriented mindsets are hypothesized to demonstrate considerable temporal stability, thereby suggesting that baseline orientations are likely to be strong predictors of subsequent orientations (Kooij et al., 2018).

Furthermore, there is a paucity of research that differentiates between general and problematic social media use, or that incorporates parental perspectives as an additional contextual lens. On the one hand, the majority of parents concur with the significance of media-related activities and competencies for the transition from school to working life. On the other hand, the dangers of distraction caused by high media consumption are emphasized (Calmbach & Schleer, 2020). It is therefore hypothesized that parental concerns primarily relate to problematic patterns of social media use, rather than to overall usage time. This is consistent with external assessments of self-regulatory difficulties and perceived risks for future development. The present study addresses these gaps by providing a longitudinal analysis of young adults’ transition from school to employment. This addresses the research gap in longitudinal approaches to future time perspective, as highlighted by Kooij et al. (2018), for example. The analysis explicitly differentiates between general and problematic social media use and examines how these patterns intersect with future-oriented transition and moratorium mindsets, as well as with parental perceptions of media-related influences on career development of their children.

Derived from the conceptual framework as well as the state of research, the present study addresses the following research questions:

- RQ1: How do parents perceive the influence of their children’s (social) media use on career opportunities during the transition from school to work?
- RQ2: To what extent do parental assessments of the influence of (social) media use on career opportunities differ in relation to young adults’ transition and moratorium orientations?
- RQ3: To what extent are general and problematic social media use associated with changes in transition-oriented and moratorium-oriented mindsets over a one-year period, after controlling for baseline orientations?

## 4. Materials and Methods

To address these matters, quantitative longitudinal data of the project “VEIF—Trajectories of Excessive Internet Use in Families” (short: VEIF) are used, which was funded by the German Research Foundation (DFG). A market research institute collected the data from t1 in 2023 and the data from t2 in 2024. In each family, one young adult and one associated parent were interviewed in a dyadic design. At t1 *N* = 443 dyads were conducted, at t2 *N* = 355. In the VEIF project, a sample with an increased risk of problematic digital media use compared to the general population was investigated. For this purpose, before the first collection of data was started in 2016, an oversampling of adolescents with an increased risk of problematic digital media use was realized (a more detailed description of the study design and the recruitment process can be found in Wartberg et al., 2017).

### 4.1. Measures

The parents’ assessment of their children’s media use on career prospects was surveyed using an open-ended question: “How do you think your child’s use of the media affects their career prospects?”. The responses were reviewed by at least two researchers to ensure intersubjective consistency and coded according to their content. An explanation of the distinguished categories is provided in Section 5.1.

The social media use of young adults was recorded in hours and minutes for a typical weekday and weekend day. These values were then utilized to calculate a common variable for average daily usage time in hours. The mean duration of these interactions was just under two hours per day (see Table 1).

For measuring problematic social media use, the Social Media Disorder Test (Wartberg et al., 2023), a shortened four-item-questionnaire adapted from an analogue scale measuring problematic online gaming (Pontes et al., 2021) was used. The items have a five-level response format (1 = “never”, 2 = “rarely”, 3 = “sometimes”, 4 = “often”, 5 = “very often”). The total score can be determined by the mean value of the given answers (range: 1 to 5). A higher score indicates a higher degree of self-assessed problematic social media use. The scale shows high reliability with a Cronbach’s alpha of 0.90 (see Table 2).

The orientation towards transition and moratorium were measured by two questionnaires developed by Reinders (2005). The scales employed both utilize a four-point response format, with the transition orientation scale ranging from (1) not at all to (4) very much, and the moratorium orientation scale ranging from (1) never to (4) often. In the former, the seven items that comprise the scale (e.g., “I have firm plans for my future, and I also believe that I will achieve them”) reflect clearer life planning and therefore a stronger focus on future time perspectives. By contrast, in the latter, which consists of ten items (e.g., “Only how you live today counts”), there is a rejection of commitment which reflects a stronger orientation on the present. The mean value for each scale was calculated (see Table 1). The reliability indices were 0.78 and 0.72 at t1 and t2 for the transition and 0.87 and 0.88 for the moratorium scale (see Table 2).

### 4.2. Sample Description

The sample comprises 443 adolescents at t1 (201 (45.1%) females and 245 (54.9%) males of the total sample) and 355 at t2 (159 (44.4%) females and 199 (55.6%) males of the total sample). Their average age was 20.11 years (SD = 0.91) at t1 and 21.11 years (SD = 0.89) at t2. In total, 73 (16.5%) of the adolescents surveyed were still in school, 4 (0.9%) did not attend school and the other 368 (83.1%) had completed their schooling at t1. Of these 368 cases, 32 (2.9% of the total sample) had graduated from school at a low education level, 209 (19.1%) at a medium education level and 126 (11.5%) at a high education level. Another person (0.1%) had left school without graduating. In the event that the young people had already completed their schooling, they were also asked about their current occupation status. Of the 368 cases, 190 (17.4%) were in vocational training or apprenticeships, 69 (6.3%) were studying, and 82 (7.5%) were in full-time employment with completed training. The remaining young adults are engaged in alternative forms of employment (13 young adults (1.3%)) or are not currently employed (14 young adults (1.3%)).

### 4.3. Statistical Analyses

The statistical software SPSS (version 29.0, IBM, 2023, New York, NY, USA) was used to calculate frequencies, means, standard deviations, reliability coefficients, multivariate one-way ANOVAs, correlation analyses and multiple linear regression analyses. The mean comparisons were calculated to compare different assessments by parents of the influence of digital media on young people’s career opportunities and their transition and moratorium orientations (Section 5.1 and Section 5.2). These findings reflect parents’ attitudes toward the influence of digital media on the transition from school to work. The multiple linear regressions were calculated with the two scales for transition and moratorium orientation as dependent variables at t2. In addition to the baseline measures of transition and moratorium orientation at t1, control variables of age, gender, school graduation and occupation status, the independent variables of daily social media usage times, the Social Media Disorder Test, and the scales for transition and moratorium orientation at t1 were utilized as predictors (Section 5.3).

## 5. Results

The results in this chapter are structured along the research questions addressing, first, the parents’ perspectives on the role of the Internet for school-to-work transition (Section 5.1), second, potential differences in the transition and moratorium orientations of young adults regarding their parents’ perspectives (Section 5.2) and, third, the influence of social media usage on transition and moratorium orientation of young adults (Section 5.3). The objective of these analyses is to examine the relationship between problematic social media use and the future time perspective of young adults including their parents’ perspectives on school-to-work transition.

### 5.1. Parents’ Perspectives on the Role of the Internet for School-to-Work Transition

The reviewing and coding of the open-ended question on how parents assess the role of the Internet for their children’s career prospects resulted in the identification of five categories. These categories are documented here with two anchor examples each: 1 = no seen impact (e.g., “no influence” or “I don’t think so.”), 2 = positive impact (e.g., “She learns a lot on the internet.” or “He uses it for further education, which is good.”), 3 = negative or critical impact (e.g., “He is neglecting school.” or “I am concerned that she is using her cell phone too much at work.”), 4 = ambivalent or depending on the context (e.g., “So far, she has managed to complete her education despite the high media presence, and she often uses it to get things done.” or “He must be able to handle it competently, but it must not degenerate into dependency or cause him to spend too much time on it.”) and 4 = no rating (e.g., “Can’t say” or “I don’t know.”). It is apparent that parents’ assessments tend not to make explicit reference to times, problematic usage patterns, or related concerns. Instead, they are more inclined to offer generalized judgments regarding their children’s media usage.

The distribution of categories is illustrated by Table 3. It indicates that the majority of the parents surveyed do not perceive any influence from the internet (37.5%), or they did not provide an assessment (19.6%). Conversely, 26.9% of parents see positive effects of their children’s media use on their career prospects. The assessments provided offer a range of reasons for this, including the increased accessibility of career information online and the recognition of existing media-related skills as a potential asset to training, studies, or future careers. 14.2% of surveyed parents perceived a negative influence, mainly concerning excessive usage time leading to the neglect of school or work tasks, or sleep deprivation. However, parents also considered posting supposedly inappropriate content on social media platforms to have a negative impact on their children’s future prospects. A mere 1.8% of respondents attributed an ambivalent or context-dependent influence of the internet on their children’s career prospects. Respondents emphasized that setting priorities when using the internet was key, and that depending on the purpose, this could result in advantages or disadvantages.

### 5.2. Differences in Parents’ Perspectives on the Role of the Internet for School-to-Work Transition and Transition and Moratorium Orientations of Young Adults

To examine whether transition and moratorium orientations (at t1) differed depending on parental assessments of the influence of media use on career opportunities, one-way ANOVAs with Bonferroni-adjusted post hoc tests were conducted. The analysis revealed significant group differences in transition orientation (see Table 4). Bonferroni-adjusted pairwise comparisons indicated that adolescents whose parents perceived media use as having a positive influence reported significantly higher transition orientation compared to those whose parents perceived no influence (*MD* = 1.47, *p* = 0.005) and those reporting a negative or critical influence (*MD* = 3.56, *p* < 0.001). Furthermore, transition orientation was significantly lower in the negative or critical influence group compared to the no influence group (*MD* = −2.09, *p* < 0.001) and the no evaluation group (*MD* = −1.90, *p* = 0.011). No significant differences emerged for the ambivalent or context-dependent group (all *p* > 0.15). Overall, the pattern suggests that a positively perceived media influence is associated with higher transition orientation, whereas a negatively perceived influence is associated with lower transition orientation.

No significant group differences were found for moratorium orientation. Neither Tukey nor Bonferroni-adjusted comparisons reached statistical significance, indicating that parental evaluations of media influence were not systematically related to moratorium orientation.

### 5.3. (Problematic) Social Media Usage and Orientation Towards Transition or Moratorium

To identify correlations between scales of transition and moratorium orientation among young adults, the correlations between relevant variables were first calculated (see Table 5). While the age of young adults shows no significant correlation with other variables, a positive correlation becomes apparent between gender and both social media use and transition orientation at t1. Regarding moratorium orientation, a negative correlation was observed at the cross-sectional and longitudinal levels. Accordingly, young men tend to be more moratorium-oriented, while young women show a stronger tendency to be transition-oriented and spend more time on social media. Graduation level was positively related to transition orientation at t1 and t2, and negatively associated with problematic social media use. Except for a small correlation with transition orientation at t1, occupation status showed only negligible correlations with the remaining variables. Prolonged social media use is associated with a higher score on the Social Media Disorder Test and a less transition-oriented perspective at t1. However, no influences could be demonstrated in the longitudinal analysis or with regard to moratorium orientation. The Social Media Disorder Test exhibits significant negative correlations with transition orientation and positive correlations with moratorium orientation in the cross-lagged as well as longitudinal perspective. Consequently, young adults with problematic social media use tend to display moratorium orientation rather than transition-oriented perspectives. There are also significant correlations between the transition and moratorium orientation scales themselves. While the correlations within the scales show a moderate positive relation over time, the small correlations between the scales are negative. (The only exception is the correlation between transition orientation at t1 and moratorium orientation at t2, which does not appear to be significant.) Therefore, both perspectives are less compatible with each other.

In a second step, longitudinal linear regression analyses were performed using the predictive variables of daily social media usage, the Social Media Disorder Test, orientation towards transition and moratorium at t1 as well as control variables age, gender, graduation level and occupation status (see Table 6). In the linear regression model, all predictors are found to be insignificant with the exception of the respective scales at t1. The resultant variance explained for the transition orientation scale is *R*^2^ = 0.23, with transition orientation identified as the only significant predictor, exhibiting a standardized beta coefficient of 0.50. As with moratorium orientation, the situation is analogous. The variance explained is *R*^2^ = 0.15, and the standardized beta coefficient of the scale for moratorium orientation at t1 is 0.37. It can be concluded that both models demonstrate low to medium variance explanation.

## 6. Discussion

The present study set out to examine the relationship between young adults’ social media use, both in terms of general and problematic engagement, and their orientation towards the future during the transition from school to work. In addition, the study sought to explore how parents perceive the impact of their children’s media use on career prospects and if their assessment reflects in transition and moratorium orientations of their children. The present study sought to contribute to a more nuanced understanding of how digital practices may be related to young people’s future time perspectives besides individual and social influencing factors in a deep mediatized society.

Regarding the parents’ assessment of media influences on their children’s school-to-work transition, the majority either did not perceive a specific influence of (social) media use on their children’s career prospects or reported positive effects (RQ 1). This phenomenon can be interpreted as indicative of a growing acceptance of digital media as an integral component of both everyday learning and vocational preparation in a deep mediatized society (Hepp, 2019). Parents frequently alluded to opportunities for acquiring information and skills, which corresponds with the view that digital technologies can serve as valuable developmental and career resources (Dennen et al., 2024; Uhls et al., 2017; Zhang et al., 2024). Conversely, a smaller group articulated concerns regarding excessive use or the potential for distraction through digital media, emphasizing apprehensions about deleterious consequences for concentration, education, or reputation. These negative perceptions are also regarded in the literature as indicative of problematic social media use (Cheng et al., 2021; Wartberg et al., 2023). These issues have the potential to exert a detrimental effect on academic and occupational performance, or to manifest as a form of escapism, a phenomenon that can prove problematic during the transition from education to employment (Díaz-López et al., 2021; Moqbel & Kock, 2018; Przepiorka & Blachnio, 2016).

The identified group differences suggest that parental perceptions of media influence are meaningfully associated with young adults’ career-related future time perspectives during the school-to-work transition (RQ 2). Specifically, a positively perceived influence of media use is linked to a stronger transition orientation. Conversely, a negatively perceived influence is associated with significantly lower transition orientation, indicating a less future-oriented and less structured approach to career development. Importantly, no group differences emerged for moratorium orientation, suggesting that parental evaluations of media engagement are more closely related to proactive, future-oriented career planning than to tendencies toward postponement or exploratory delay. These findings underscore that the subjective meaning attributed to social media use—rather than media engagement per se—may play a relevant role in shaping young adults’ future-oriented career mindsets (e.g., Senekal et al., 2023).

Cross-sectional and longitudinal analyses demonstrated that problematic social media use was negatively associated with transition orientation and positively related to moratorium orientation (RQ 3). These findings lend support to the assumption that maladaptive digital engagement may coincide with reduced self-regulation and difficulties in sustaining goal-directed, future-oriented behavior. Within the theoretical framework of future time perspectives, such patterns can be interpreted as indicative of a diminished capacity to organize present actions in accordance with long-term goals. Concurrently, no substantial relationships were identified for general social media use, suggesting that the extent of usage is less consequential than the quality and regulation of engagement for developmental functioning or functional usage patterns for the school-to-work transition. This finding aligns with the observations of Dennen et al. (2024), which underscored the multifaceted nature of social media utilization during the transition to university. In the longitudinal regression analyses, however, problematic social media use did not predict subsequent changes in transition or moratorium orientation once initial levels were controlled for. The strongest predictors for both outcomes were the corresponding baseline orientations, indicating substantial temporal stability over the one-year interval. This finding suggests that mindsets oriented towards the future and moratoriums represent relatively stable developmental dispositions, rather than being easily influenced by behavioral factors such as media use as well as individual factors such as school graduation or current occupation status. Nevertheless, the significant cross-sectional and longitudinal associations indicate the presence of concurrent interactions between media-related self-regulation and future time perspectives, which merit further examination over extended time periods.

The findings of this study highlight the contradictory role of social media in the transition of young people from education to employment. Whilst general engagement can provide resources for identity development and career planning, problematic use patterns have been linked to less structured and less future-oriented mindsets in a cross-lagged and longitudinal perspective. In accordance with research emphasizing the mediating role of self-regulation in digital contexts, the results indicate that the developmental impact of social media is contingent not on usage duration, but rather on the quality and purpose of engagement (Dennen et al., 2024; Senekal et al., 2023). Parental perceptions, reflecting both normalization and concern, remain an important contextual factor in understanding how young adults negotiate digital opportunities and risks during their transition to adulthood.

The present study provides further insights into the field, extending the current state of research. However, it is important to note that the study is not without its limitations. The results were collated in a sample of young adults, for which no representative sample was obtained. Youths who reported experiencing difficulties with regard to digital media use were deliberately oversampled (for further details on the sampling strategy, see Wartberg et al., 2017). Additionally, future research could apply structural equation modeling to integrate measurement and structural models. Moreover, in the context of problematic digital media use in young adulthood, in addition to self-ratings, the approach of working with external assessments (frequently parental judgments, see, for example Berber et al., 2025) has been employed. However, in the present study, this additional source of information was not incorporated. A number of other constructs have been empirically associated with transition and moratorium orientation. In particular, the focus is on social factors and individual factors such as mental state and well-being (e.g., Kooij et al., 2018). However, these issues were not addressed in the present study and should be integrated as an inclusive model in further research. Furthermore, a significant variation was observed in the responses of parents with regard to their evaluation of their children’s media usage in relation to their future career prospects. A number of the responses were concise, comprising merely a small number of words. In contrast, others were extensive, containing several sentences and necessitating interpretation by the researchers. Despite the efforts of at least two researchers to categorize the statements, a certain degree of bias remains.

## 7. Conclusions

The present study makes a significant contribution to the emerging field of research that explores the nexus between future time perspectives, social media engagement, and the school-to-work transition in emerging adulthood. Drawing on longitudinal data from the VEIF-project, the analyses provide a differentiated understanding of how young people’s digital practices, parental perceptions, and orientations towards transition or moratorium interrelate.

The influence of social media on the career development of young adults is perceived differently by parents, depending on whether their offspring use social media in a problematic way. A salient finding that merits further deliberation is the shift in parental perception regarding the prospects offered by digital media during the transition to working life. Cross-sectional and longitudinal analyses revealed that problematic social media use is associated with a reduced transition orientation and an increased moratorium orientation, while social media use in general has no significant influence. This suggests that maladaptive media patterns may be associated with less goal-directed, future-oriented planning. However, longitudinal regression analyses did not confirm the predictive effects of social media use or problematic engagement on future orientations one year later. This finding suggests that problematic social media use, while it may be associated with a less future-oriented mindset, does not necessarily determine developmental trajectories over time. It has been demonstrated that, in contrast, the most effective predictor is individual stability in transition and moratorium orientations. This suggests a notable degree of stability in relation to orientation, with a relatively distinct focus on future time perspectives.

In accordance with the findings of preceding research emphasizing the significance of self-regulation and future time perspective in digital contexts, these results suggest that the developmental significance of social media is less dependent on usage time and more dependent on the quality and regulation of the individual’s own social media engagement as well as individual and social influencing factors on future time perspectives. The findings of this study emphasize the necessity for a more nuanced perspective on digital media use in emerging adulthood and its influence on the transition from education to employment. This perspective must acknowledge both the potential of digital media as a developmental resource and the risks associated with problematic use during the transition from school to work. Consequently, the utilization of digital media for the purpose of information and resource management can undoubtedly facilitate the development of future time perspectives among young adults as they transition into the working world.

## Figures and Tables

**Table 1:** Descriptive data of the used variables.

Variables	Sample (*N_t_*_1_ = 443 Dyads; *N_t_*_2_ = 355 Dyads) M (SD)
Daily Social Media Usage (in hours) (t1)	1.95 (1.54)
Social Media Disorder Test (t1)	1.44 (0.68)
Orientation towards Transition (t1)	20.18 (3.63)
Orientation towards Transition (t2)	20.30 (3.49)
Orientation towards Moratorium (t1)	24.24 (5.92)
Orientation towards Moratorium (t2)	24.06 (6.28)

**Table 2:** Reliability Coefficients of the used scales (Cronbach’s Alpha).

	t1	t2
Social Media Disorder Test	0.90	-
Orientation towards Transition	0.78	0.72
Orientation towards Moratorium	0.87	0.88

**Table 3:** Percentages of the coded parental responses.

Answer	Sample (*N* = 443 Parents) *N* (Percentage)
No seen impact	166 (37.5%)
Positive impact	119 (26.9%)
Negative or critical impact	63 (14.2%)
Ambivalent or depending on the context	8 (1.8%)
No rating	87 (19.6%)

**Table 4:** Bonferroni-Adjusted Pairwise Comparisons for Transition Orientation (t1).

Comparison	Mean Difference (I–J)	SE	95% CI
Positive vs. no seen impact	1.47 ^2^	0.42	[0.29, 2.65]
Positive vs. negative or critical impact	3.56 ^3^	0.54	[2.03, 5.10]
Positive vs. no rating	1.66 ^2^	0.49	[0.27, 3.05]
No seen vs. negative or critical impact	−2.09 ^3^	0.52	[−3.56, −0.63]
Negative or critical impact vs. no rating	−1.90 ^1^	0.58	[−3.54, −0.27]

Notes: Only statistically significant Bonferroni-adjusted comparisons are displayed. ^1^ *p* < 0.05, ^2^ *p* < 0.01, ^3^ *p* < 0.001. CI = confidence interval.

**Table 5:** Correlation matrix.

	1	2	3	4	5	6	7	8	9	10
(1) Age (t1)	-									
(2) Gender (t1)	0.00	-								
(3) Graduation Level (t1)	0.10	−0.06	-							
(4) Occupation Status (t1)	0.01	−0.01	−0.04	-						
(5) Social Media Use (t1)	−0.04	0.17 ^3^	−0.05	0.06	-					
(6) Social Media Disorder Test (t1)	0.06	−0.08	−0.21 ^3^	0.08	0.21 ^3^	-				
(7) Orientation towards Transition (t1)	−0.01	0.12 ^1^	0.27 ^3^	−0.11 ^1^	−0.13 ^2^	−0.35 ^3^	-			
(8) Orientation towards Transition (t2)	0.04	0.08	0.15 ^1^	0.01	−0.08	−0.16 ^2^	0.46 ^3^	-		
(9) Orientation towards Moratorium (t1)	0.07	−0.11 ^1^	−0.09	0.04	0.07	0.17 ^3^	−0.17 ^3^	−0.13 ^1^	-	
(10) Orientation towards Moratorium (t2)	−0.05	−0.14 ^2^	0.01	0.08	0.03	0.15 ^2^	−0.07	−0.11 ^1^	0.41 ^3^	-

Notes: ^1^ *p* < 0.05, ^2^ *p* < 0.01, ^3^ *p* < 0.001.

**Table 6:** Results of the Longitudinal Linear Regression with parameters conducted at t1.

Parameter	Orientation Towards Transition (t2) Standardized Beta Coefficients	Orientation Towards Moratorium (t2) Standardized Beta Coefficients
Age (t1)	0.04	−0.07
Gender (t1)	−0.01	0.09
Graduation Level (t1)	0.03	0.08
Occupation Status (t1)	0.07	0.04
Daily Social Media Usage (t1)	−0.00	0.01
Social Media Disorder Scale (t1)	0.05	0.09
Orientation towards Transition (t1)	0.50 ^3^	-
Orientation towards Moratorium (t1)	-	0.37 ^3^
**Corrected R^2^**	**0.23**	**0.15**

Notes: independent variables collected at first survey wave; ^3^ *p* < 0.001.

## Data Availability

The data presented in this study are available on reasonable request to research-mediaeducation@fau.de.
